# Molecular characterization of breast cancer cell pools with normal or reduced ability to respond to progesterone: a study based on RNA-seq

**DOI:** 10.1186/s43141-023-00541-6

**Published:** 2023-08-08

**Authors:** Mariana Bustamante Eduardo, Irene Keller, Nathalie Schuster, Stefan Aebi, Rolf Jaggi

**Affiliations:** 1 Department for BioMedical Research, University of Bern, Bern, Switzerland; 2 Department of Surgery, Feinberg School of Medicine, Northwestern University, Chicago, USA; 3 Swiss Institute of Bioinformatics, Lausanne, Switzerland; 4 Department of Medical Oncology, Cantonal Hospital, Lucerne, Switzerland

**Keywords:** Breast cancer, Estrogen receptor alpha, Progesterone receptor, Gene expression, RNA sequencing, CRISPR/Cas9

## Abstract

**Background:**

About one-third of patients with estrogen receptor alpha (ERα)-positive breast cancer have tumors which are progesterone receptor (PR) negative. PR is an important prognostic factor in breast cancer. Patients with ERα-positive/PR-negative tumors have shorter disease-free and overall survival than patients with ERα-positive/PR-positive tumors. New evidence has shown that progesterone (P4) has an anti-proliferative effect in ERα-positive breast cancer cells. However, the role of PR in breast cancer is only poorly understood.

**Methods:**

We disrupted the PR gene (*PGR*) in ERα-positive/PR-positive T-47D cells using the CRISPR/Cas9 system. This resulted in cell pools we termed PR-low as P4 mediated effects were inhibited or blocked compared to control T-47D cells. We analyzed the gene expression profiles of PR-low and control T-47D cells in the absence of hormone and upon treatment with P4 alone or P4 together with estradiol (E2). Differentially expressed (DE) genes between experimental groups were characterized based on RNA-seq and Gene Ontology (GO) enrichment analyses.

**Results:**

The overall gene expression pattern was very similar between untreated PR-low and untreated control T-47D cells. More than 6000 genes were DE in control T-47D cells upon stimulation with P4 or P4 plus E2. When PR-low pools were subjected to the same hormonal treatment, up- or downregulation was either blocked/absent or consistently lower. We identified more than 3000 genes that were DE between hormone-treated PR-low and control T-47D cells. GO analysis revealed seven significantly enriched biological processes affected by PR and associated with G protein-coupled receptor (GPCR) pathways which have been described to support growth, invasiveness, and metastasis in breast cancer cells.

**Conclusions:**

The present study provides new insights into the complex role of PR in ERα-positive/PR-positive breast cancer cells. Many of the genes affected by PR are part of central biological processes of tumorigenesis.

**Supplementary Information:**

The online version contains supplementary material available at 10.1186/s43141-023-00541-6.

## Background

About 70% of breast cancers express the steroid hormone receptor estrogen receptor alpha (ERα), a member of the steroid hormone receptor family. Growth and proliferation of ERα-positive tumors depend on endogenous estrogens, and they are considered good candidates for endocrine therapies. Although the progesterone receptor (PR), another member of the steroid hormone receptor family, is an ERα-regulated gene, about a quarter of ERα-positive breast cancers are PR negative [1, 2]. Several studies have shown that patients with ERα-positive tumors that lack the PR have shorter disease-free and overall survival than patients with ERα-positive/PR-positive breast cancers [3–5].

PR is an important prognostic marker in ERα-positive breast cancer [6]. However, its role is rather complex and controversial [7]. As a member of the steroid hormone receptor family, PR regulates gene transcription in response to ligand binding [8]. On the one hand, PR has been described to be a player in breast tumorigenesis, but on the other hand, PR has been described to have an antiproliferative role under estrogenic conditions in breast cancer cells. In line with the pro-tumorigenic role, progestogen treatment induced the expansion of cancer stem cells [9, 10] and promoted cell proliferation in vitro [11, 12]. Additionally, the inclusion of progestogens as part of menopausal hormone therapy (MHT) increased the risk of developing breast cancer [13]. However, this was only true for some specific synthetic progestogens such as medroxyprogesterone acetate (MPA), norethindrone (NETA), and levonorgestrel, and not for naturally occurring progestogen, progesterone (P4) [14, 15]. In line with an antiproliferative role of PR, ligand-activated PR can modulate the activity of ERα, thereby diminishing tumor growth [16–18]. In fact, there is an interest in using progestogens in combination with endocrine therapy to improve the survival of ERα-positive/PR-positive breast cancer patients [19]. Interestingly, progestin treatment has been shown in the past to improve the survival of breast cancer patients [20, 21]. The ratio of the two PR isoforms, PR-A and PR-B, may be predictive for progestin and antiprogestin response. Breast cancers with high PR-A/PR-B ratios metastatic growth was inhibited by antiprogestins, and the metastatic growth of breast cancers with low PR-A/PR-B ratios was inhibited with progestins [22, 23].

There is a need to better understand the role of PR and the implications of its absence in ERα-positive breast cancer. In this study, we modulated the expression of PR in the ERα-positive/PR-positive T-47D cell line using the CRISPR/Cas9 system and generated cell pools with normal or reduced ability to respond to P4. We identified differentially expressed (DE) genes and pathways that were affected by a reduced response to P4. We discuss how genes and pathways may be relevant for the characteristic phenotype of ERα-positive/PR-positive and ERα-positive/PR-negative tumors.

## Material and methods

### Cell culture

The human breast cancer cell line T-47D (HTB-133™) was purchased from American Type Culture Collection (ATCC). Cell culture reagents were purchased from Gibco (Thermo Fisher Scientific). T-47D cells were cultured in Dulbecco’s Modified Eagle Medium (DMEM) supplemented with 10% fetal bovine serum (FBS), 1% penicillin/streptomycin, and 200-mM L-glutamine. Cells were passaged three times a week at a split ratio of 1:2. Five days before starting hormone treatments, the medium was replaced by phenol red-free DMEM supplemented with 10% charcoal-stripped FBS [24], 1% penicillin/streptomycin, and 200-mM L-glutamine. Cells were regularly screened for the absence of mycoplasma contamination using a PCR Mycoplasma Test Kit I/C (Promokine). The T-47D cell line was authenticated by STR profiling (Microsynth, Balgach, Switzerland).

### Reduction of functional PR using the CRISPR/Cas9 system

The CRISPR/Cas9 genome editing tool was used to disrupt the *PGR* gene in T-47D cell line. Single guide RNAs (sgRNAs) were designed against exon 1 of the *PGR* gene (Supplementary Fig. 1a) using three bioinformatic tools: CRISPR design tool [25], DNA2.0 gRNA design tool [26], and CCTop [27]. Each tool ranked candidate sgRNAs based on the combination of their on-target and off-target ranks. The best sgRNAs according to at least two of the three bioinformatic tools were tested in vitro using the Guide-it Complete sgRNA Screening system (Takara Clontech) (Supplementary Fig. 1b) according to the manufacturer’s instructions with minor modifications. Briefly, a 1018-bp fragment of the *PGR* gene containing all sgRNA target sites was amplified using the Q5® High-Fidelity 2 × Master Mix (New England BioLabs) and primers outside the sgRNA-targeted regions (forward: 5′-CCTGGACGGGCTACTCTTC-3′ and reverse: 5′-CCTTCCTCCTCCTCCTTTA-3′). The 1018-bp fragment of the *PGR* gene, each in vitro transcribed sgRNA and recombinant Cas9 nuclease were incubated at 37 °C for 1 h. Cleavage products were analyzed on an Agilent 2100 Bioanalyzer using the DNA 1000 reagent kit (Agilent Technologies). Five sgRNA oligos targeting both PR isoforms that efficiently cleaved the *PGR gene *in vitro (sgRNA1x: CCAGTGAAGCCGTCTCCGC, sgRNA2: GTGGATGAAATCCATCACCG, sgRNA3: CAGGACGCGCCGATGGCGCC, sgRNA5: TGAGAGCCCTCACTGGTCCG, and sgRNA11x: CCCCGCTCATGAGCCGGTC) and one sgRNA oligo directed against the luciferase gene (sgRNALUC1: CTTCGAAATGTCCGTTCGGT) were annealed and cloned into the pSpCas9(BB)-2A-GFP vector (Addgene plasmid no. 48138; the vector was a gift from Feng Zhang) as described elsewhere [28]. The GFP expressed from this vector was later used to sort cells that express Cas9 and gRNAs (see below). Combinations of two (sgRNA1x and sgRNA2, or sgRNA3 and sgRNA11x) or five (sgRNA1x, sgRNA2, sgRNA3, sgRNA5 and sgRNA11x) constructs coding for sgRNAs were transfected in order to increase the chances of disrupting *PGR* (as gene editing may generate indels on more than one region of the gene and deletions of larger regions between two sgRNAs are also possible). For control cells, T-47D cells were transfected with one construct coding for the sgRNA directed against the luciferase gene. For transfection, cells were seeded into 6-cm plates, and transfections were performed using Lipofectamine 3000 (Thermo Fisher Scientific) according to manufacturer’s instructions. Between 30,000 and 68,000 GFP-positive cells were sorted 2 days after transfection using a FACS ARIA III (BD Bioscience) and placed back in culture in 24-well plates. Cells were grown until four confluent 6-cm plates were obtained (after 4 to 6 weeks). Three control pools and seven pools transfected with sgRNAs against the *PGR* gene were obtained, each from independent transfections.

### Screening of cell pools

DNA was extracted from cell pools using the QIAamp DNA Blood kit (Qiagen). A 1018-bp fragment of the *PGR* gene was amplified with primers outside the target regions of the sgRNAs (forward: 5′-CCTGGACGGGCTACTCTTC-3′ and reverse: 5′-CCTTCCTCCTCCTCCTTTA-3′) using Q5® High-Fidelity 2 × Master Mix (New England BioLabs). The PCR products were analyzed on an Agilent 2100 Bioanalyzer (Supplementary Fig. 1c).

### Luciferase reporter assay

The level of functional PR was measured in cell pools using a luciferase reporter assay (2 × PRE-TK-luc, addgene plasmid no. 11350). The vector was a gift from Donald McDonnell [29], and it contains two copies of a consensus progesterone response element (PRE) and a thymidine kinase (TK) promoter driving the luciferase reporter (firefly). Cell pools were transfected in triplicates and 24 h after transfection, they were treated with 100-nM water-soluble P4 (Sigma-Aldrich) or vehicle (water) for 24 h. Cells were then lysed and mixed with Luciferase assay reagent (Promega). Luciferase was measured on a Spark 10 M plate reader (Tecan).

### RNA extraction

Cell pools were treated with 100 nM P4, 100 nM P4, plus 10 nM-β-estradiol (E2) (Sigma-Aldrich) or vehicle (water and 100% ethanol) for 8 h. Cells were then lysed and RNA extracted as described previously [30]. RNA yield was assessed using a NanoDrop ND-1000 Spectrophotometer (NanoDrop Technologies) and a Qubit 2.0 fluorometer (Invitrogen). RNA quality was assessed on an Agilent 2100 Bioanalyzer using the RNA 6000 Nano Kit (Agilent Technologies).

### Quantitative reverse transcription PCR (RT-qPCR)

Hormone-responsive genes (*GREB1*, *SERPINA3*, *DUSP1*, and *SCUBE2*), breast-cancer-related genes (*ESR1*, *PGR*, *GATA3*, and *RAB31*), and control genes (*RLPL0*, *GUSB*, and *UBB*) were measured by RT-qPCR on an Applied Biosystems ViiA 7 Real-Time PCR system (Applied Biosystems). RT-qPCR was carried out using 5 ng of RNA, TaqMan gene expression assays (Applied Biosystems), and the SuperScript III One-Step RT*-*PCR System with Platinum Taq DNA Polymerase (Invitrogen). For the fold change analysis, the 2^-delta-delta CT was calculated as described elsewhere [31]. For this, gene of interest levels were normalized relative to the mean expression of the three control genes (delta CT). The difference between treated and untreated gave the delta-delta CT.

### RNA-seq sample preparation and sequencing

Between 200 ng and 1 µg of high-quality RNA (RIN > 8) from three independent T-47D control (vehicle, P4 and P4/E2 treated) and three independent pools transfected with sgRNAs against the *PGR* gene (vehicle, P4 and P4/E2 treated) were used for cDNA synthesis and library preparation using the TruSeq-stranded mRNA Sample Preparation kit (Illumina). The cDNA libraries were multiplexed in nine samples per lane and sequenced on an Illumina HiSeq 3000 using 100-bp single-end sequencing.

### RNA-seq analysis

Between 30.5 and 58.8 million reads were obtained per sample. The reads were aligned to the human genome version GRCh38 using Hisat v.2.1.0, and the number of reads overlapping the genes in the Ensembl annotation build 89 was counted with FeatureCounts from Subread v.1.5.3. Gene expression levels were compared among experimental groups using the R-package DESeq2 v.1.18.1 [32]. *P*-values were corrected for multiple testing based on the Benjamini-Hochberg (BH) procedure, and a significance threshold of 0.05 was applied.

### Gene ontology enrichment analysis

Gene Ontology (GO) enrichment analysis was performed using the topGO: Enrichment Analysis for Gene Ontology, R package version 2.32.0.[33]. Terms were ranked based on *P*-values from a Fisher’s exact test using the weight01 algorithm to account for the hierarchical structure of the GO. The 30 most significant terms from each GO subontology (biological process, molecular function, and cellular component) were retained. The REVIGO tool [34] was used for visualizing enriched GO terms. Cytoscape v.3.6.0 was used to visualize interactive graphs produced by REVIGO.

### Statistical analysis and visualization

Statistical analyses were performed, and graphs were constructed using GraphPad Prism v.7.01 or R v.3.4.2. Venn diagrams were produced using the BioVenn website [35].

## Results

### Generation and selection of cell pools with normal or reduced ability to respond to P4

**Fig. 1:**
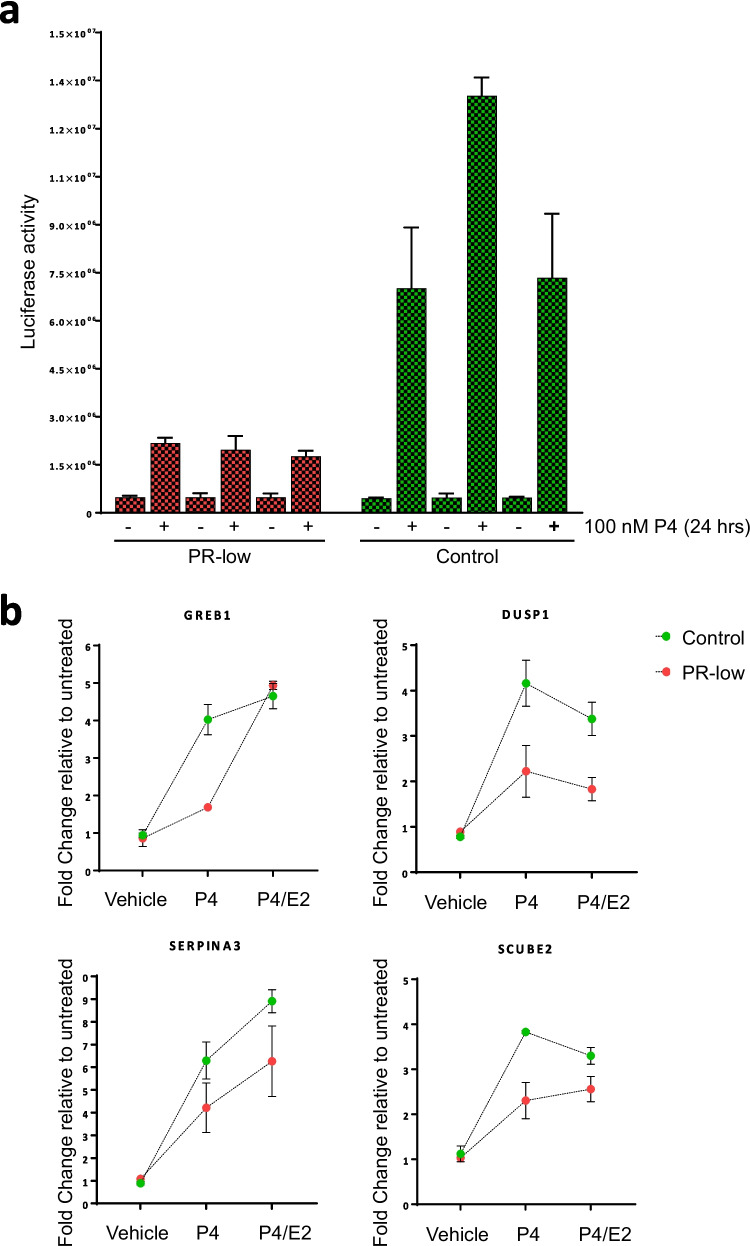
Characterization of T-47D control cells and PR-low. **a** PR function was assessed in the three PR-low (red) and three control (green) pools with a PRE-driven luciferase construct (2 × PRE-TK-luc). Each treatment was done in triplicate. Error bars represent the SEM. **b** Endogenous progesterone (P4)-responsive genes were measured in T-47D control cells (green) and PR-low pools by RT-qPCR. Samples were measured in triplicates. P4 and P4/estradiol (E2) treatment was for 8 h. Fold change was calculated relative to untreated as described in Material and methods. Error bars represent the SEM

We used the CRISPR/Cas9 system to disrupt the *PGR* gene in ERα-positive/PR-positive T-47D breast cancer cells. To test the specific effects of P4 and P4 plus E2 (P4/E2), cells were grown in phenol red-free medium supplemented with hormone-depleted FBS. To indirectly measure the level of functional PR, we measured exogenous and endogenous P4-responsive genes. First, cells were transiently transfected with a 2 × PRE-TK promoter-luciferase reporter and treated with P4 or vehicle for 24 h. The average fold induction of luciferase activity after P4 treatment was 20 in T-47D control cells and four in pools transfected with sgRNAs against *PGR* (Fig. 1a). Second, cells were treated with vehicle, P4, and P4/E2 for 8 h, the RNA was extracted, and endogenous hormone-responsive genes (Fig. 1b), control genes. and breast cancer-related genes (not shown) were measured by RT-qPCR. The induction of hormone-responsive genes was higher in T-47D control cells compared to pools transfected with sgRNAs against *PGR* (Fig. 1b). The latter had a reduced capacity to induce exogenous (luciferase reporter assay) and endogenous hormone-responsive genes compared to T-47D control cells; thus, we named them PR-low pools. Three PR-low pools with the lowest induction of hormone-responsive genes (not shown) were selected for RNA-seq.

### RNA-seq analysis of T-47D control and PR-low

**Fig. 2:**
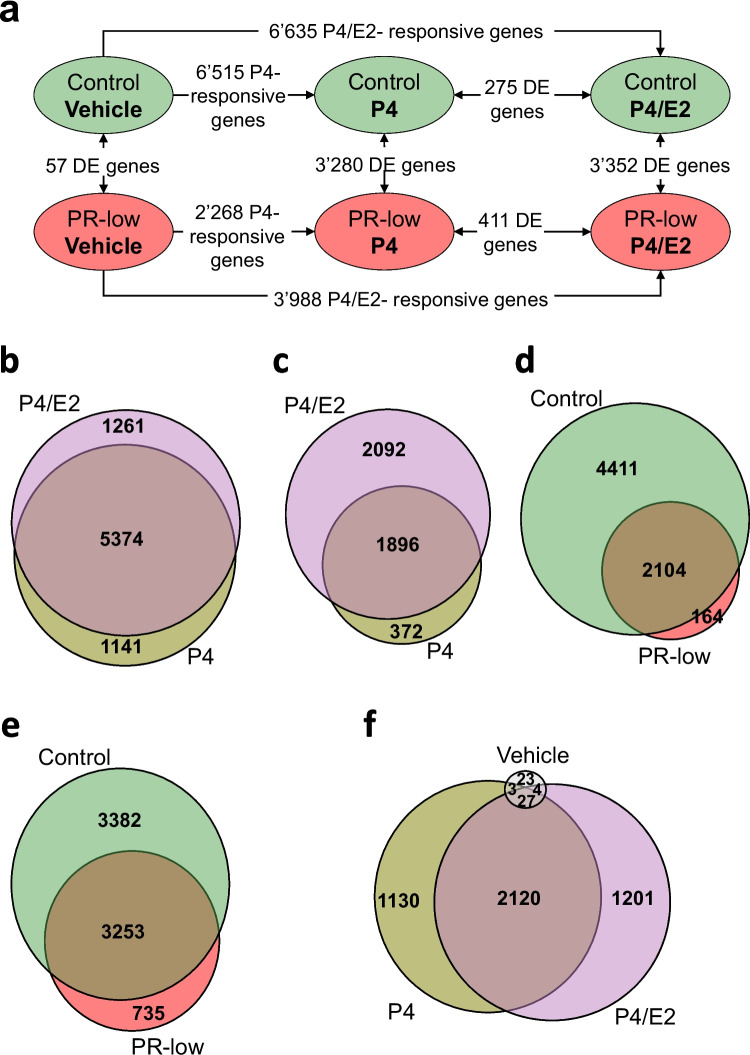
Differential expression analyses of RNA-seq samples. **a** Differentially expressed (DE) genes (BH-adjusted *P* < 0.05) between T-47D control cells (green) and PR-low pools (red) and among treatments. The data for control and PR-low are mean values of three independent biological replicates. The number of DE genes is shown for each comparison. Venn diagrams representing overlaps of DE genes between P4-responsive and P4/E2-responsive genes in control (**b**), P4-responsive and P4/E2-responsive genes in PR-low (**c**), P4-responsive genes in control and P4-responsive genes in PR-low (**d**), P4/E2-responsive genes in control and P4-responsive genes in PR-low (**e**), and DE genes between control and PR-low after vehicle, P4 and P4/E2 treatments (**f**). P4, progesterone; E2, estradiol; PR, progesterone receptor

We searched for differentially expressed (DE) genes (Benjamini-Hochberg (BH)-adjusted *P* < 0.05) among treatments (vehicle vs. P4, vehicle vs. P4/E2, and P4 vs. P4/E2) using DESeq2 in T-47D control cells and in PR-low pools (Fig. 2a). We identified 6515 and 2268 P4-responsive genes in T-47D control cells and PR-low pools, respectively (Supplementary Tables 1 and 2). Also, we identified 6635 and 3988 genes modulated by P4/E2 in T-47D control cells and PR-low pools, respectively (Supplementary Tables 3 and 4). We observed a big overlap between P4- and P4/E2-responsive genes in T-47D control cells, about 82% of P4-responsive genes were modulated in the same direction by P4/E2, and about 81% of P4/E2-responsive genes were modulated in the same direction by P4 alone (Fig. 2b). In PR-low, about 82% of P4-responsive genes were modulated in the same direction by P4/E2, and about 47% of P4/E2-responsive genes were modulated in the same direction by P4 in T-47D control cells (Fig. 2c).

Similarly, we searched for DE genes between T-47D control cells vs. PR-low pools for each treatment. We identified 57 DE genes between vehicle-treated T-47D controls cells and vehicle-treated PR-low pools, 3280 DE genes between P4-treated T-47D controls cells and P4-treated PR-low pools, and 3352 DE genes between P4/E2-treated T-47D controls cells and P4/E2-treated PR-low pools (Fig. 2a, Supplementary Tables 5–7). About 64% of DE genes between P4-treated T-47D controls cells and P4-treated PR-low pools overlapped with DE genes between P4/E2-treated T-47D controls cells and P4/E2-treated PR-low pools (Fig. 2f).

### Response to P4 in T-47D control and PR-low

**Fig. 3:**
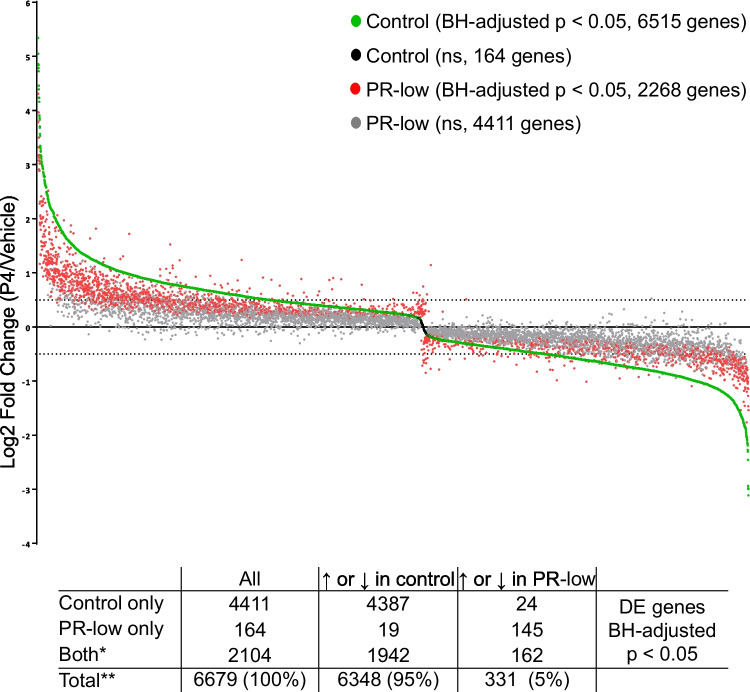
Differentially expressed (DE) genes (BH-adjusted *P* < 0.05) in T-47D control cells and PR-low pools after progesterone (P4) treatment. Genes are aligned along the *x*-axis according to their log2 fold change (P4/vehicle) in T-47D control cells from highest to lowest (green if significant and black if not significant). The same arrangement of the genes on the *x*-axis was used for the corresponding PR-low pools (red if significant and grey if not significant). Shown are mean values of three independent biological replicates. About 95% of genes in PR-low pools were less responsive to P4 treatment than control cells resulting in nonsignificant regulation (4411 genes in gray). Induction/repression of PR-responsive genes was still significant in 2104 genes (red) as compared to controls (6515 genes in green). The number of differentially expressed genes in control and PR-low is depicted in the table (inset). ↑Higher, ↓lower, ns stands for not significant, *significant in control and PR-low, **significant in control and/or PR-low. PR, progesterone receptor

To confirm that PR-low pools had a lower induction of P4-responsive genes, we compared the P4-responsive genes in T-47D control cells to the P4-responsive genes in PR-low pools. From the 6515 genes modulated by P4 in T-47D control cells, a total of 4411 genes (66%) were not significantly modulated in PR-low pools. About 93% of P4-reponsive genes in PR-low pools overlapped with genes modulated by P4 in T-47D control cells (Fig. 2d). and they were induced or repressed to lower levels in PR-low as compared to T-47D control cells (Fig. 3). These comparisons show that a large majority of P4-regulated genes in PR-low pools was less responsive or not responsive to P4.

### Functional classification of P4/E2-responsive genes

To explore the action of P4 in estrogenic conditions, we analyzed P4/E2 response in both T-47D control cells and PR-low pools. We focused on P4/E2 treatment because there is a functional crosstalk between PR and ERα [16], and P4/E2 treatment better recapitulates physiological hormonal scenarios in T-47D cells than P4 alone since breast cancer cells in patients are also exposed simultaneously to both hormones. Treatment of cells with P4/E2 resulted in significant modulation of 6635 and 3988 genes in T-47D control cells and PR-low pools, respectively. We observed an overlap of 3253 genes modulated by P4/E2 in T-47D control cells and PR-low pools (Fig. 2e). *CHRM*, *SEC14L2*, *TRIM22*, *SGK1*, *RBP2*, *ZBTB16*, *TMEM63C*, *HSD11B2*, *FAM105A*, and *AZGP1P1* were among the most strongly induced genes, and *CXCR4*, *AMIGO2*, *PCED1B*, *P2RY10*, *PTHLH*, *NDP*, *HDAC9*, *CAPN5*, *PPARGC1A*, and *NR2F1* were among the top repressed genes in T-47D control cells (Supplementary Table 3). Based on the P4/E2-responsive genes in T-47D control cells and PR-low pools, we performed a Gene Ontology (GO) term enrichment analysis for biological process, molecular function, and cellular component (Supplementary Tables 8 and 9). In both PR-low pools and T-47D control cells, we identified biological processes related to apoptosis, cell migration, and DNA replication among the 30 most significantly enriched terms. In addition, we identified biological processes related to tRNA export from nucleus and epithelial cell morphogenesis only in control T-47D cells and biological processes related to G protein-coupled receptor signaling pathway, cell cycle, and cell growth only in PR-low pools.

### GO enrichment analysis of DE genes between control and PR-low

**Fig. 4:**
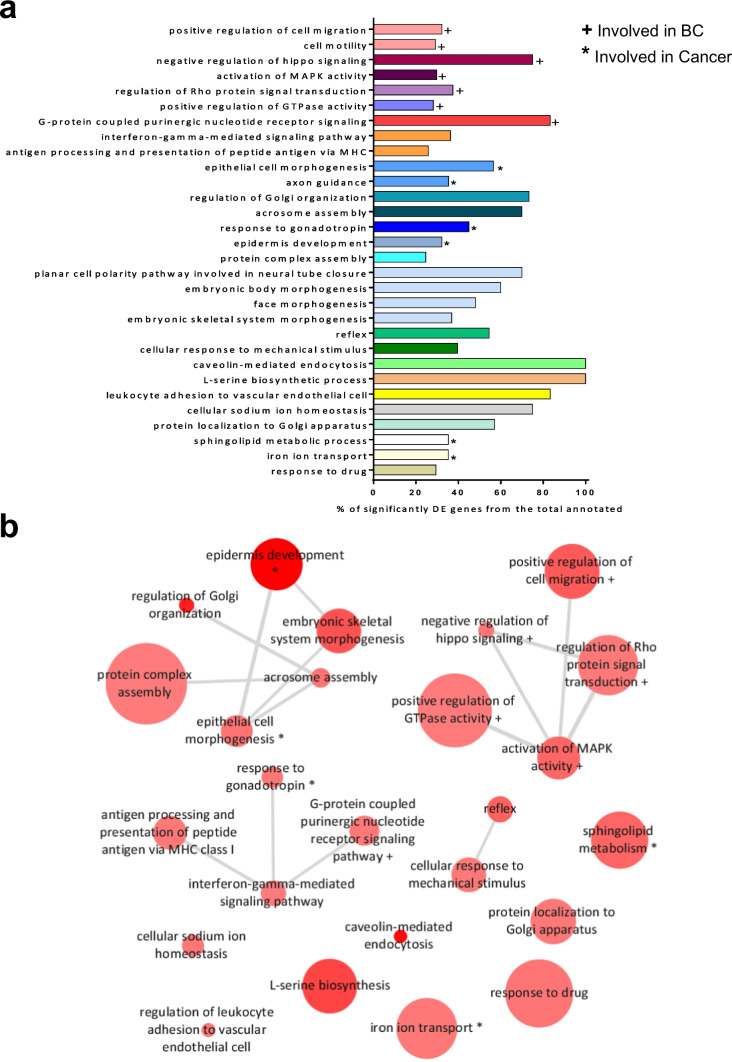
Gene Ontology (GO) term enrichment analysis. Biological processes enriched for differentially expressed (DE) genes between P4/E2-treated T-47D control cells and P4/E2-treated PR-low pools. **a** The 30 most significantly deregulated biological processes (*P*-value < 0.01). For each GO annotation, the % of significantly DE genes from the total number of annotated genes is depicted. REVIGO [34] was used to assess the functional redundancy among the biological processes; redundant GO annotations are shown in the same color. + Involved in breast cancer. *Involved in cancer. **b** Interactive graph created by REVIGO, redundant GO annotations are removed. The sizes of the circles indicate the relative frequencies of GO terms in the underlying GO annotation database (UniProt) (frequency = percentage of annotated human proteins in UniProt with a GO term). Darker shading indicates lower *p*-values; similar GO terms are connected by lines, with thicker lines indicating closer similarity; the length of the lines is arbitrary. + Involved in breast cancer. *Involved in cancer. BC, breast cancer

Only 57 genes were DE between untreated PR-low pools and T-47D control cells, while when hormone-treated T-47D control cells and hormone-treated PR-low pools were compared, we identified more than 3000 DE genes. This shows that the main difference between T-47D control cells and PR-low pools is the level of P4 response. We used the 3352 DE genes between P4/E2-treated T-47D control cells and P4/E2-treated PR-low pools to identify biological processes, molecular functions, and cellular components affected by P4 (Supplementary Table 10). GO enrichment analysis revealed 13 biological processes previously associated with cancer among the top 30. Seven of them have previously been associated with breast cancer: positive regulation of cell migration, activation of MAPK activity, cell motility, negative regulation of hippo signaling, G protein-coupled purinergic nucleotide receptor signalling pathway, regulation of Rho protein signal transduction, and positive regulation of GTPase activity (Fig. 4a–b).

## Discussion

At present, the role of PR and the implications of its absence in ERα-positive breast cancer remain to be characterized. Using the CRISPR/Cas9 system, we generated T-47D cells with normal (T-47D control cells) and reduced (PR-low pools) response to P4. Our PR-low cell pools represented a valuable model for studying the response to P4 in this ERα-positive breast cancer cell line. Two-thirds of P4-responsive genes identified in T-47D control cells were not induced or repressed upon P4 treatment in PR-low pools. In the remaining 33% of P4-responsive genes, induction or repression was attenuated. Gene expression in PR-low pools and T-47D control cells was astonishingly similar in the absence of hormones: only 57 genes were DE between PR-low pools and T-47D control cells. Therefore, P4 response was characterized in more detail by analyzing P4/E2-responsive genes and by comparing P4/E2-resposive genes in T-47D control cells vs. P4/E2-resposive genes in PR-low pools.

We identified 6515 P4-responsive genes in T-47D control cells. Among them were many known targets of PR [36–39]. Similar studies reported a lower number for progestin (R5020)-responsive genes in T-47D cells: 1287 genes after 3 h of treatment [36] and 3248 genes after 2 to 12 h treatment [37]. About 73% and 42% of the genes reported by Kougiomutzi et al. [36] and Singhal et al. [37], respectively, overlapped with the P4-responsive genes we identified (data not shown). Various differences in the experimental design, technology, and thresholds in data analysis may explain many of the differences. For instance, Kougioumtzi et al. and Singhal et al. deprived the cells of steroids 24 and 48 h before hormone treatment, respectively, while we performed hormone treatments after 5 days of steroid deprivation. Kougioumtzi et al. used a microarray technology, while Singhal et al. used RNA-seq.

Many of the hormone-responsive genes we identified were also reported in the literature, and they may be of potential interest in the context of ERα-positive breast cancer. The top downregulated gene after P4/E2 treatment in T-47D control cells was *CXCR4*. This gene was also among the top downregulated genes after P4 treatment, and its expression was significantly higher in PR-low pools when compared to T-47D control cells. In addition, this gene was involved in two (activation of MAPK activity and cell motility) of the seven biological processes we identified to be affected by P4 and associated with breast cancer. When *CXCR4* expression was evaluated using the the Cancer Gene-Expression Miner -bc-GenExMiner v.4.8 tool [40], we observed that its expression was significantly higher in ERα-positive/PR-negative tumors when compared to ERα-positive/PR-positive tumors (*P* < 0.0001). CXCR4 is a G protein-coupled chemokine receptor which has a very well-known role in breast cancer proliferation and metastasis [41–44]. The inhibition of CXCR4 reduced growth, migration, and invasion in mouse models carrying breast cancer [45–48], and the anti-tumor effect of PARP1 inhibitors was also enhanced when CXCR4 was inhibited [47]. CXCR4 may be of potential interest since P4/E2 and P4 treatment resulted in the downregulation of this gene. Interestingly, E2 treatment alone enhanced the expression of *CXCR4* in ERα-positive breast cancer cell lines [49]. Here, we emphazise the observation that *CXCR4* is regulated by E2 and P4 in opposite directions. It may be reasonable to speculate that *CXCR4* downregulation is part of the putative protective role of P4, while in the absence of PR or PR activation, the upregulation of *CXCR4* may contribute to tumorigenesis, progression, and metastasis.

We analyzed DE genes between vehicle and P4/E2 treatments in T-47D control cells and PR-low pools. We focused on P4/E2 treatment conditions and not on P4 alone as breast cancer cells are usually exposed to both hormones in patients and numerous functional cross talks exist between PR and ERα in vivo [16]. Among the 6635 DE genes modulated by P4/E2 in T-47D control cells, we identified numerous known P4-responsive genes [36–39], E2-responsive genes [50], and genes responsive to P4 and E2 [36, 50, 51]. GO term enrichment analysis revealed biological processes related to DNA replication, tRNA export from nucleus, cell migration, epithelial cell morphogenesis, and apoptosis among the most deregulated processes. Our results are analogous to those obtained by Mohammed et al. [16] and Finlay-Schultz et al. [17], who compared progestogen/E2 treatment to E2 alone in ERα-positive/PR-positive breast cancer cells (MCF-7 and T-47D) and patient derived tumor xenografts, respectively. They identified enriched pathways associated with apoptosis, cell death, and epithelial cell morphogenesis [16, 17]. All three studies showed that progestogen/E2 treatment of ERα-positive/PR-positive breast cancer cells or xenografts resulted in the enrichment of pathways which reflected an antiproliferative role of PR. Contrariwise, P4/E2 treatment in PR-low pools resulted in the enrichment of biological processes associated with cell cycle and cell growth.

We took advantage of our model to explore the P4 response in estrogenic conditions by comparing P4/E2-treated T-47D control cells and P4/E2-treated PR-low pools. GO enrichment analyses revealed that 13 out of the 30 most enriched biological processes have been previously described to be associated with cancer. We considered that seven of them may be of special interest as they have been described before in the context of breast cancer [48, 52–60]; they all are affected by P4 and relate to G protein-coupled receptor (GPCR) signaling pathways. GPCR-mediated signaling has been implicated in growth regulation, tumor initiation, tumor progression, invasiveness, and metastasis [52, 57, 61–67].

## Conclusion

We identified genes and biological processes affected by P4 in the presence of E2 which have been previously described in the context of breast cancer. For instance, the GPCR receptor, CXCR4 may be relevant in ERα-positive/PR-negative breast cancer. It remains important to further explore the role of PR in the context of breast cancer and to find more effective therapies for patients with ERα-positive/PR-negative cancer which still have a significantly worse prognosis than patients with ERα-positive/PR-positive tumors.

### Supplementary Information

**Additional file 1:** **Supplementary Fig. 1.** Design, selection and screening of sgRNAs. a) Schematic representation of the *PGR *gene showing the pre-selected sgRNAs targeting exon 1 of isoform B only (blue box) or isoforms A and B (purple box). The red lines denote the Cas9 cleavage site. Grey arrows represent the forward primer at position 161 and the reverse primer at position 1159 used for amplifying the *PGR* gene for in vitro tests and screenings. The black line below the *PGR* gene represents the PCR product of about 1018 bp. b, Bioanalyzer gel image showing the in vitro testing of pre-selected sgRNAs (not all shown). Five sgRNAs were selected (*) based on their efficiency to cleave a synthetic target fragment of *PGR*. sgRNAs representing 19 mers (x) and 20 mers were tested. c, Bioanalyzer gel image showing the amplification of the *PGR* gene in control and PR-low pools. The expected size of the intact wild-type (WT) allele is 1018 bp. PCR products smaller than 1018 bp correspond to the expected sizes when 2 sgRNAs result in the removal of the fragment between them leading to larger deletions and shorter PCR products. The size of bands was determined by the Bioanalyzer software. In PR-low pools bands at around 1018 bp may correspond to intact WT allele and/or allele with indel mutations. Three control pools and PR-low pools were selected for RNA-seq (‡).

**Additional file 2:** **Supplementary Table 1.** Differentially expressed (DE) genes (BH-adjusted *P* < 0.05) between vehicle treated T-47D control cells and P4-treated T-47D control cells. Three independent biological replicates per treatment/condition were sequenced. **Supplementary Table 2.** Differentially expressed (DE) genes (BH-adjusted *P* < 0.05) between vehicle-treated PR-low pools and P4-treated PR-low pools. Three independent biological replicates per treatment/condition were sequenced. **Supplementary Table 3.** Differentially expressed (DE) genes (BH-adjusted *P* < 0.05) between vehicle treated T-47D control cells and P4/E2-treated T-47D control cells. Three independent biological replicates per treatment/condition were sequenced. **Supplementary Table 4.** Differentially expressed (DE) genes (BH-adjusted *P* < 0.05) between vehicle-treated PR-low pools and P4/E2-treated PR-low pools. Three independent biological replicates per treatment/condition were sequenced. **Supplementary Table 5.** Differentially expressed (DE) genes (BH-adjusted *P* < 0.05) between vehicle-treated PR-low pools and vehicle treated T-47D control cells. Three independent biological replicates per treatment/condition were sequenced. **Supplementary Table 6.** Differentially expressed (DE) genes (BH-adjusted *P* < 0.05) between P4-treated PR-low pools and P4-treated T-47D control cells. Three independent biological replicates per treatment/condition were sequenced. **Supplementary Table 7.** Differentially expressed (DE) genes (BH-adjusted *P* < 0.05) between P4/E2-treated PR-low pools and P4/E2-treated T-47D control cells. Three independent biological replicates per treatment/condition were sequenced. **Supplementary Table 8.** Top 30 gene ontology (GO) biological processes, cellular components (CC) and molecular functions (MF) significantly enriched after P4/E2 treatment in control pools. Annotated = total number of expressed genes in the term; significant = number of genes with BH-adjusted *P-*value < 0.05 in the term. *P*-values are from a Fisher’s exact test run in TopGO using the weight01 algorithm to account for the GO hierarchy. **Supplementary Table 9.** Top 30 gene ontology (GO) biological processes, cellular components (CC) and molecular functions (MF) significantly enriched after P4/E2 treatment in PR-low pools. Annotated = total number of expressed genes in the term; significant = number of genes with BH-adjusted *P*-value < 0.05 in the term. *P*-values are from a Fisher’s exact test run in TopGO using the weight01 algorithm to account for the GO hierarchy. **Supplementary Table 10.** Top 30 gene ontology (GO) biological processes, cellular components (CC) and molecular functions (MF) significantly enriched among the 3′352 differentially expressed genes between P4/E2 treated control and PR-low pools. Annotated = total number of genes in the term; significant = number of genes with BH-adjusted *P*-value < 0.05 in the term. *P-*values are from a Fisher’s exact test run in TopGO using the weight01 algorithm to account for the GO hierarchy.

## Data Availability

RNA-seq data have been deposited in the European Nucleotide Archive (ENA) at EMBL-EBI under accession number PRJEB42823.

## Abbreviations

BH: Benjamini-Hochberg
DE: Differentially expressed
E2: Estradiol
ER*α*: Estrogen receptor alpha
GO: Gene ontology
GPCR: G protein-coupled receptor
PR: Progesterone receptor
PRE: Progesterone response element
P4: Progesterone
sgRNA: Single guide RNA
