# Supplementary material for: Molecular characterization of breast cancer cell pools with normal or reduced ability to respond to progesterone: a study based on RNA-seq
Source: J Genet Eng Biotechnol. 2023 Aug 8;21:81. doi: 10.1186/s43141-023-00541-6 (PMC10406740; doi:10.1186/s43141-023-00541-6)
Supplement: Supplementary file 1 — Additional file 1: Supplementary Fig. 1. Design, selection and screening of sgRNAs. a) Schematic representation of the PGR gene showing the pre-selected sgRNAs targeting exon 1 of isoform B only (blue box) or isoforms A and B (purple box). The red lines denote the Cas9 cleavage site. Grey arrows represent the forward primer at position 161 and the reverse primer at position 1159 used for amplifying the PGR gene for in vitro tests and screenings. The black line below the PGR gene represents the PCR product of about 1018 bp. b, Bioanalyzer gel image showing the in vitro testing of pre-selected sgRNAs (not all shown). Five sgRNAs were selected (*) based on their efficiency to cleave a synthetic target fragment of PGR. sgRNAs representing 19 mers (x) and 20 mers were tested. c, Bioanalyzer gel image showing the amplification of the PGR gene in control and PR-low pools. The expected size of the intact wild-type (WT) allele is 1018 bp. PCR products smaller than 1018 bp correspond to the expected sizes when 2 sgRNAs result in the removal of the fragment between them leading to larger deletions and shorter PCR products. The size of bands was determined by the Bioanalyzer software. In PR-low pools bands at around 1018 bp may correspond to intact WT allele and/or allele with indel mutations. Three control pools and PR-low pools were selected for RNA-seq (‡). [file 43141_2023_541_MOESM1_ESM.pdf]

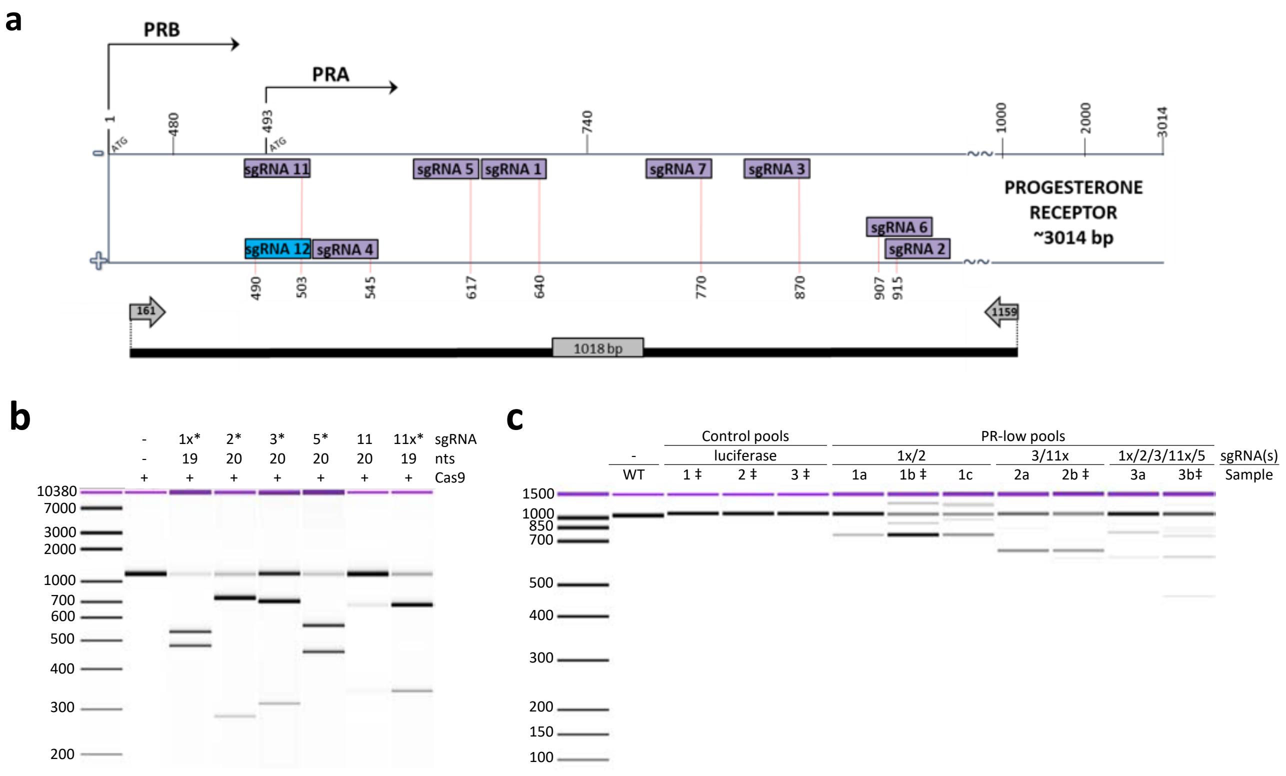

SUPPLEMENTARY FIGURE 1. **<u>Design, selection and screening of sgRNAs</u>. a)** Schematic representation of the *PGR* gene showing the pre-selected sgRNAs targeting exon 1 of isoform B only (blue box) or isoforms A and B (purple box). The red lines denote the Cas9 cleavage site. Grey arrows represent thethe forward primer at position 161 and the reverse primer at position 1’159 used for amplifying the *PGR* gene for *in vitro* tests and screenings . The black line below the *PGR* gene represents the PCR product of about 1’018 bp. **b,** Bioanalyzer gel image showing the *in vitro* testing of pre-selected sgRNAs (not all shown). Five sgRNAs were selected (*) based on their efficiency to cleave a synthetic target fragment of *PGR*. sgRNAs representing 19-mers (x) and 20-mers were tested. **c,** Bioanalyzer gel image showing the amplification of the *PGR* gene in control and PR-low pools. The expected size of the intact wild-type (WT) allele is 1’018 bp. PCR products smaller than 1018 bp correspond to the expected sizes when 2 sgRNAs result in the removal of the fragment between them leading to larger deletions and shorter PCR products. The size of bands was determined by the Bioanalyzer software. In PR-low pools bands at around 1’018 bp may correspond to intact WT allele and/or allele with indel mutations. Three control pools and PR-low pools were selected for RNA-seq (‡).
